# Single Nucleotide Polymorphisms in *TLR4* Affect Susceptibility to Tuberculosis in Mexican Population from the State of Veracruz

**DOI:** 10.1155/2020/2965697

**Published:** 2020-01-29

**Authors:** Enrique Ortega, Sujhey Hernández-Bazán, Beatriz Sánchez-Hernández, Ileana Licona-Limón, Javier Fuentes-Dominguez

**Affiliations:** ^1^Department of Immunology, Instituto de Investigaciones Biomédicas, Universidad Nacional Autónoma de México, Ciudad Universitaria, CP 04510 CDMX, Mexico; ^2^Department of Genetics, Instituto Nacional de Ciencias Médicas y de la Nutrición Salvador Zubirán, CDMX, Mexico; ^3^Program of Micobacteriosis. Servicios de Salud de Veracruz, Xalapa, Veracruz, Mexico

## Abstract

Tuberculosis is still a global public health problem, with an estimated 10 million new cases and 1.6 million deaths in 2017. Of all humans infected with *M. tuberculosis*, only 10-15% will develop active tuberculosis disease during their lifetime, and data suggest that along with environmental factors, genetic factors influence susceptibility to develop active disease. Toll-like receptors (TLRs) are pattern recognition receptors that play a central role in the initiation and shaping of adaptive immune responses, and several TLRs have been shown to recognize mycobacterial components. In this work, we performed a case-control study to determine if common single nucleotide polymorphisms (SNPs) in genes encoding TLRs 1, 2, 4, 6, and 10 are associated with susceptibility to develop active tuberculosis in population from the state of Veracruz, Mexico. The study included 279 cases and 569 controls. The results show that the frequency of two SNPs in TLR4 was significantly higher in controls than in tuberculosis patients. The minor allele (G) of rs4986790 in *TLR4* (D299G) decreased the risk of active tuberculosis in the allelic (A vs. G, OR = 0.31, 95%CI = 0.09‐0.81, *p* = 0.01) and in the dominant genetic model (AA vs. GG+AG, OR = 0.26, 95%CI = 0.09‐0.77, *p* = 0.02). Similarly, the minor allele (T) of rs4986791 in *TLR4* (T399I) decreased the risk of active disease in the allelic model (C vs. T, OR = 0.29, 95%CI = 0.10‐0.90, *p* = 0.03). We did not find an association of SNPs in *TLR1* (N248S), *TLR2* (R753Q), *TLR6* (S249P), and *TLR10* (A153S and V298I) with tuberculosis disease. These results suggest that in this population, genetic variants of *TLR4* affect the susceptibility for suffering active tuberculosis disease.

## 1. Introduction

Tuberculosis (TB), an infectious disease usually caused by *Mycobacterium tuberculosis* (Mtb), is a global public health problem, with an estimated 10 million new cases of TB and 1.6 million deaths in 2017 [1]. Despite significant advances in its detection and treatment, TB remains the leading cause of death by a single infectious agent worldwide [1].

In humans, infection with Mtb can result in the development of active TB disease or in an immune response that can maintain the infection in a latent state (>85% of infected individuals). Under conditions causing immunosuppression, latent TB can progress to active disease. Many factors affect the outcome of the initial infection and determine the progression from latent infection to active disease. In addition to factors such as age, gender, microbial infections, chronic diseases, and environmental aspects, there is strong evidence that genetic factors are important in determining the individual susceptibility to develop active TB [2].

Several studies in different human populations have identified many candidate genes in which polymorphisms can affect susceptibility or resistance to develop TB disease (reviewed in [2, 3]). Among them, genes encoding Toll-like receptors (TLRs) have received particular attention because of the central role of these receptors in the initiation and shaping of an adaptive immune response, and various TLRs have been shown to recognize mycobacterial components [4, 5]. TLRs are a group of pattern recognition receptors that play a key role in the immune system by detecting pathogens and initiating a signaling cascade that results in the secretion of inflammatory cytokines, type I IFN, chemokines, and antimicrobial peptides. In turn, these mediators orchestrate an inflammatory reaction and recruit and activate macrophages and other effector cells of the innate immune response. TLRs also contribute to the activation and maturation of dendritic cells that are essential for the initiation and for shaping the adaptive T cell response [6].

Various TLRs have been shown to recognize mycobacteria and their extracellular products. Thus, TLR2-TLR1 and TLR2-TLR6 heterodimers, as well as TLR4, have been shown to recognize mycobacterial PAMPs and mediate activation of dendritic cells and macrophages [7–10]. TLR10, on its part, serves as a modulatory receptor with inhibitory properties on TLR2-derived immune responses. Single nucleotide polymorphisms (SNPs) in TLRs can alter ligand-receptor interactions or modulate receptor signaling and thus can influence susceptibility or resistance to diseases. It is therefore not surprising that several studies have looked for an association between genetic polymorphisms in TLRs and susceptibility to TB in several human populations. However, results are in many instances inconsistent and inconclusive, emphasizing the need for more studies analyzing larger numbers of samples from different human populations.

In this study, we investigated the association of polymorphisms in the genes for *TLR1*, *TLR2*, *TLR4*, *TLR6*, and *TLR10*, with susceptibility to TB in a population of the Mexican state of Veracruz, a state with a relatively high prevalence of TB (27.4 cases/100,000 people) [11]. Our results found that two common SNPs in *TLR4* that are in strong linkage disequilibrium confer a protective effect to develop active TB in this population.

## 2. Materials and Methods

### 2.1. Study Population

A total of 279 TB cases and 569 healthy controls were included in this case-control association study. All participants, as well as their parents and grandparents, were born in Mexico. We consider participants in this study as Mexican mestizos, i.e., having both European and Amerindian ancestry, as only indigenous groups living in very remote and isolated areas of Mexico can be considered to have pure indigenous ancestry. All participants in this study have a Spanish-derived last name and spoke Spanish, and a small proportion also spoke an indigenous language. All participants were older than 18 years and were living in three regions of the state of Veracruz, México: Coatzacoalcos (31 cases, 71 controls), Poza Rica (156 cases, 298 controls), and Veracruz (92 cases, 200 controls). TB patients were diagnosed with pulmonary TB by clinical symptoms and at least one of the following: sputum smear examinations for acid-fast bacilli, or a positive culture for *Mycobacterium tuberculosis*, or chest X-ray. All patients have been diagnosed as TB patients by the medical staff of the Health Services of the state of Veracruz, Mexico, and were receiving anti-TB medication. Patients coinfected with human immunodeficiency virus (HIV), or having diabetes mellitus or an autoimmune disease, were excluded from the study. Healthy controls were unrelated individuals with no history and no suggestive symptoms of TB, living in the same area and similar socioeconomic conditions as patients. Written informed consent was obtained from all patients and controls. This study was reviewed and approved by the Committee for Research in Humans of the Instituto de Investigaciones Biomédicas, UNAM, and by the Committee for Research, Bioethics, and Biosafety of the Health Services of the state of Veracruz, México.

### 2.2. Blood Samples and DNA Isolation

Blood samples were collected from May 2011 through June 2013. From each participant, samples of 4 ml of venous blood were collected directly into EDTA-containing tubes (BD Vacutainer®, Franklin Lakes, NJ, USA). Genomic DNA was extracted from blood samples following standard protocols [12]. DNA from each sample was quantified in a NanoDrop 1000 Spectrophotometer (Thermo Scientific, Wilmington, DE, USA) and was kept frozen at -20°C until used.

### 2.3. Gene Polymorphism Genotyping

Seven nonsynonymous single nucleotide polymorphisms (SNPs) in five genes were investigated in all participants: *TLR1* (N248S; rs4833095), *TLR2* (R753Q; rs5743708), *TLR4* (D299G; rs4986790 and T399I; rs4986791), *TLR6* (S249P; rs5743810), and *TLR10* (A163S; rs11466649 and V298I; rs11466651). These SNPs were selected based on previous reports of their association with TB and because these polymorphisms have been reported to have functional consequences.

SNP genotyping was performed using validated predesigned TaqMan™ SNP Genotyping Assays (Life Technologies/Applied Biosystems, Foster City, CA) according to the manufacturer's instructions. All assays were carried out on an ABI 7500 HT Real-time PCR System. The TaqMan call rates for genotyping were over 99%.

### 2.4. Statistical Analysis

Wilcoxon 2-sample rank-sum test was used to compare median ages of cases and controls. The first step of the statistical analysis was to compare the distribution of demographic characteristics and allelic and genotypic frequencies between cases and controls using the *χ*^2^ test, Fisher's exact test, and univariate unconditional logistic regression. In this crude analysis, TLR4 polymorphisms, age, sex, speaking an indigenous language, and educational level were associated with TB infection risk (Table 1). Odds ratios (OR) and 95% confidence intervals (CI) for genetic associations were assessed by using univariate and multivariate unconditional logistic regression (LR), with adjustment for age, sex, rural dwelling, speaking an indigenous language, and educational level (Table 2). All analyses were performed using Stata 12 (StataCorp, College Station, TX, USA). Hardy-Weinberg equilibrium (HWE) was evaluated in controls using goodness-of-fit *χ*^2^ tests within each SNP. The *D*′ values of LD plots were produced using the Haploview 4.2 program. Since this was an exploratory study, we did not introduce a correction for multiple comparisons [13]. Statistical significance was defined as *p* < 0.05.

## 3. Results

We used a case-control population study design to evaluate whether SNPs in genes for various TLRs were associated with an increased risk to develop active TB disease in Mexican adults from the state of Veracruz. All individuals participating in the study were descendants of Mexican parents and grandparents. Demographic characteristics of TB cases and controls are shown in Table 1. The distribution of age, personal habits such as smoking and alcohol consumption, and conditions such as having direct contact with patient(s) and residence in a rural area showed no statistically significant differences between cases and controls (*p* > 0.05). There was a significant difference between the proportion of females and males among groups (*p* < 0.001). The higher proportion of males in the patient group is expected as the male : female ratio among adult TB patients is approximately 2 : 1 [1]. The control group had a higher proportion of females, although this result is very probably biased because of the sampling, as persons accompanying the patient to the clinic that were recruited to participate in the study were mainly females, either wives of male patients or female in-law or friend of female patients. Other individual circumstances such as speaking an indigenous language (*p* < 0.001) and very low educational level (none+complete elementary school vs. complete secondary school+complete high school+higher) (*p* < 0.001) were identified as risk factors for TB disease in our study population.

### 3.1. Association of SNPs in TLR Genes with Active Tuberculosis

The allele and genotype frequencies of the *TLR1*, *TLR2*, *TLR4*, *TLR6*, and *TLR10* polymorphisms investigated are shown in Table 2. The genotype frequency distribution of all the seven TLR SNPs was consistent with the Hardy-Weinberg equilibrium in the control group. To determine if any of these polymorphisms could be a risk or a protective factor for developing active TB disease, we determined the odds ratios (OR). The allele and genotype frequencies of the *TLR1* (rs4833095), *TLR2* (rs5743708), *TLR6* (rs5743810), and *TLR10* (rs11466649 and rs11466651) polymorphisms were found to be similar among TB cases and controls in our study population (Table 2).

In the case of the polymorphisms in *TLR4*, the results show that the overall frequencies of the minor alleles of both SNPs D299G (rs4986790) and T399I (rs4986791) were low in the study population, as the allelic frequencies of the variant allele were only 2.8% for *TLR4* (D299G) and 2.3% for *TLR4* (T399I) in the control population. The minor allele (G) of rs4986790 in *TLR4* is associated with a decreased risk of TB disease in the study population (A vs. G, OR = 0.31, 95%CI = 0.09‐0.81, *p* = 0.01). In the dominant genetic model, the G allele is also associated with decreased risk of TB (AA vs. GG+AG, OR = 0.26, 95%CI = 0.09‐0.77, *p* = 0.02). Similarly, the minor allele (T) of rs4986791 in *TLR4* (T399I) was also found to be associated with a decreased risk of TB (C vs. T, OR = 0.29, 95%CI = 0.10‐0.90, *p* = 0.03). All significant results were adjusted by gender, speaking an indigenous language, and educational level (Table 2). Our results suggest that the variant allele (G) in the polymorphism TLR4 D299G (A/G) and the variant allele (T) in polymorphism TLR4 T399I (C/T) on *TLR4* gene confer a protective effect against active TB in this population.

Since the polymorphisms in *TLR1* (N248S), *TLR2* (R753Q), *TLR6* (S249P), and *TLR10* (A163S and V298I) are located in chromosome 4, we performed an analysis to determine if different haplotypes could be associated with an increased risk of active TB. The polymorphism in *TLR2* (R753Q) was not included in the haplotype analysis because of the very low frequency found in the population studied (overall frequency 1/1696 alleles). The results are shown in Table 3. None of the five different haplotypes present in the population showed a significant association with TB. Of the four theoretically possible haplotypes generated by the polymorphisms in the *TLR4* gene (D299G (A/G) and T399I (C/T)) located in chromosome 9, only two haplotypes are present in the population studied. This is consistent with the fact that these two SNPs have been reported to cosegregate [14]. The haplotype AC (299D/399T) is the most frequent one and is associated with an increased risk of active TB (OR = 3.51, *p* = 0.006), whereas the less frequent haplotype GT (299G/399I) is associated with a diminished risk of developing the active disease (OR = 0.35, *p* = 0.043) (Table 3).

**Figure 1:**
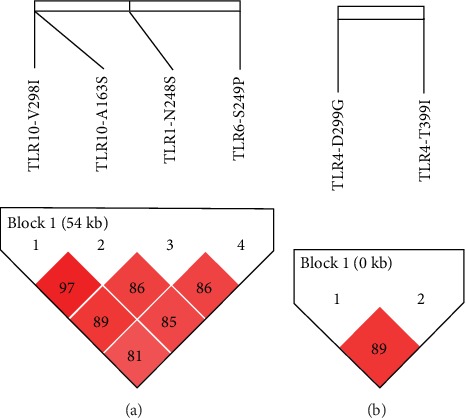
Haploview plot illustrating the linkage disequilibrium (LD) of TLR variants in the Mexican population from the state of Veracruz. (a) Haplotype-based association plot of the I298V, A163S (*TLR10*), S248N (*TLR1*), and S249P (*TLR6*) variants with TB disease. (b) Linkage disequilibrium plot of D299G and T399I SNPs from the TLR4 gene in the TB patients. The degree of pairwise LD (*r*^2^) is shown in each block.

Figure 1 shows the graphical representation of linkage disequilibrium between the four SNPs of TLR genes in chromosome 4 (*TLR1*, *TLR6*, and two in *TLR10*) and the two SNPs in *TLR4* in chromosome 9. The *TLR2* polymorphism R753Q was excluded from this analysis because of its insignificant expression in the study population. As reflected by the *D*′ values observed, both SNPs in *TLR10* are in strong linkage disequilibrium (*D*′ = 97), and LD for the other two polymorphisms studied are lower (89 > *D*′ > 81). As has been found in different populations, the two polymorphisms in *TLR4* (T399I and D299G) are in strong LD in the study population.

## 4. Discussion

TB remains a worldwide health problem, and several lines of reasoning indicate that along with environmental and bacterial factors, genetic factors of the host contribute to susceptibility for suffering active TB. Thus, to identify genes involved, many studies have been conducted to uncover the influence of polymorphisms in different immune response genes in the individual susceptibility to TB. Several polymorphisms in various genes have been shown to be associated with an increased risk of developing active TB, including genes of the innate immune response (reviewed in [3]). However, as expected for a multifactorial disease, different and sometimes contradictory results have been reported in distinct populations, emphasizing the need for additional studies analyzing larger numbers of subjects from diverse populations, as the effect of variations in one gene could differ according to the overall genetic background of the population.

Toll-like receptors are pattern recognition receptors of great relevance in the initiation and shaping of the adaptive immune response. Thus, the effects of genetic variation in TLRs have been studied in the context not only of infectious diseases but also in autoimmune and inflammatory diseases as well [15]. Different TLRs expressed in antigen-presenting cells can detect both pathogen- and danger-associated molecular patterns and induce a signaling cascade for activation of these cells for secretion of cytokines and the expression of costimulatory molecules that shape, both quantitatively and qualitatively, the activation of T cells. We analyzed single nucleotide polymorphisms in various TLRs genes, for its association with the risk for developing active TB in population from the state of Veracruz, Mexico. The state of Veracruz has a relatively high prevalence of TB (27.4 cases/100,000 people), ranking 8^th^ among the 32 states in Mexico, where the overall rate was 17.3 TB cases/100,000 people in 2016 [11]. Thus, it is important to identify factors which affect the susceptibility to TB in this population. The SNPs that we analyzed were chosen because some of them have shown an association with TB in different populations. The studied SNPs are located in *TLR1* (rs4833095), *TLR2* (rs5743708), *TLR4* (rs4986790 and rs4986791), *TLR6* (rs5743810), and *TLR10* (rs11466649 and rs11466651). TLR1-TLR2 and TLR2-TLR6 heterodimers, as well as TLR4, have been shown to recognize mycobacterial PAMPs and mediate activation of dendritic cells and macrophages [7–10]. TLR10 is still an orphan receptor, as no ligands for it have been described, although it has been shown to have a role in some infectious diseases [16] and to modulate responses mediated by TLR2 [17].

The SNP rs4833095 in *TLR1* is a nonsynonymous polymorphism that results in an asparagine to serine change of residue 248 in the extracellular domain of the receptor (N248S). This change has been proposed to affect both ligand binding and the ability to form heterodimers with TLR2, and thus, it has been studied in association with the risk of developing TB. It has been described that PBMCs from healthy controls expressing TLR1-248N exhibited an increased secretion of TNF in response to Mtb lysates and that HEK cell lines transfected with *TLR1*-248N showed an increased activation of NF-*κ*B in response to stimulation with Mtb [18]. In the same study, TLR1-248N SNP was found to be associated with protection from TB in a cohort from India. Several studies of the association of this SNP with TB susceptibility have been reported in different populations [19, 20]. In a recently published meta-analysis, Schurz et al. analyzed six studies that investigated this polymorphism and found a protective effect of the TLR1 248N variant (T allele) in both the heterozygous comparison (TC vs. CC: OR = 0.77, *p* = 0.0031) and in a dominant model (TT+TC vs. CC: OR = 0.78, *p* = 0.0021) [21]. The meta-analysis comprises populations from Asian, African, European, and Hispanic origin. The results of Ma et al. in a Hispanic population living in the USA (included in the meta-analysis) showed a tendency towards a protective effect of the T allele (248N) in the dominant model but without statistical significance (OR = 0.73, *p* = 0.22) [22]. We found no tendency of association of this polymorphism with susceptibility to TB in the studied population. The allelic frequency of the minor allele in our control population was 44.7%, very similar to the frequency in the patients' group (45.7%). Thus, it is possible that while in other populations variations in this SNP are relevant for susceptibility for TB, in a genetic background of American mestizos, such as our population and the Hispanic group of Ma et al. [22], the protective effect of the minor allele (coding for the 248N variant) could be countervailed by other genetic factors.

The SNP rs5743708 in *TLR2* results in an arginine to glutamine substitution in position 753 of the cytoplasmic portion of the receptor (R753Q). This change has been proposed to affect signaling by this receptor [23]. Although this is a rare polymorphism, several studies have reported the association of the variant allele A (753Q) with an increased risk for tuberculosis. Thus, Guo and Xia recently published the results of a meta-analysis of studies on the association of TLR2 (R753Q) with TB [24]. The analysis included 22 studies in Caucasian and Asian populations. They found a significant relationship between the allele A (753Q) and TB disease in the allelic genetic model (A vs. G, OR: 2.801, 95% CI: 2.130-3.683, *p* < 0.001), as well as in the homozygous model (AA vs. GG, OR: 5.795, 95% CI: 1.982-16.941, *p* = 0.001), heterozygous model (AG vs. GG, OR: 2.628, 95% CI: 1.888-3.569, *p* < 0.001), dominant genetic model (AA+AG vs. GG, OR: 2.786, 95% CI: 2.003-3.877, *p* < 0.001), and recessive genetic model (AA vs. AG+GG, OR: 5.568, 95% CI: 1.907-16.255, *p* = 0.002). In subgroup analysis based on ethnicity, significance was observed in both the Caucasian and Asian groups. Similarly, in a different meta-analysis, association of this SNP with increased risk for tuberculosis was found in the Asian population [25] but not in the white population. The meta-analysis of Schurz et al. [21] also analyzed studies of this polymorphism. While the global analysis showed no association, the subgroup analysis by ethnicity revealed the association of the A allele (753Q) with susceptibility in the Asian group, while in the Hispanic population (3 studies), it conferred protection against TB disease. However, not all studies have been able to show this association in Asian populations, as in a hospital-based case-control study in Chinese population, no association of this SNP with TB was detected [26]. We found only one mutant allele for this polymorphism in our study population (from a total of 1696 alleles). The frequency of this polymorphism in our population is close to zero. This result agrees with a previous report in a Mexican population in which analyzing 90 cases of TB and 90 controls, they found no single A allele [27].

The D299G (rs4986790) and T399I (rs4986791) SNPs in *TLR4* are among the most studied genetic variants of all TLRs, and their possible association with TB has been analyzed in various populations. Different results have been found in different cohorts, with some studies reporting significant associations [28–31] and others reporting no association. Distinct meta-analyses of published studies have been reported [21, 32]. Tian et al. [32] included six case-control studies in their analysis, involving 1587 controls and 2110 patients from diverse ethnicities (Caucasian, African American, Hispanics, and subjects from India and Colombia). Overall, no significant associations (all *p* > 0.05) were found between the D299G and T399I SNPs in *TLR4* gene and TB. Schurz et al. [21] included in their meta-analysis twelve studies of the D299G SNP and found no significant association with TB. They also analyzed nine studies of T399I, again finding no significant association with TB in the overall analysis. In the subgroup analysis, however, they found that in the Asian population (4 studies), the minor allele (T) and the TC and TT genotypes (in the T399I polymorphism) were associated with increased susceptibility to TB. In contrast, we found that in our study population, the minor allele G in *TLR4* (299G) and the minor allele T in *TLR4* (399I) were less frequent in patients than in controls, and thus, these polymorphisms were significantly associated with decreased susceptibility to TB (Table 2; *TLR4* 299G OR = 0.31, *p* = 0.01; *TLR4* 399I OR = 0.29, *p* = 0.03). The opposite effects of *TLR4* 299G and 399I variants on susceptibility to TB between our population and the Asian subgroup of Schurz et al. [21] illustrate, as has been found for TLR2 (R753Q) (see above), that the same SNP could show different effects in different studies depending on several factors, including sample size and power of the study, differences in criteria for definition of cases, and genetic differences among study populations.

Two previous studies have analyzed the possible association of polymorphism *TLR4* (D299G) with TB in populations from México. Torres-Garcia et al. [27] analyzed 90 patients with TB and 90 controls from the state of Oaxaca and did not detect an association, while Rosas-Taraco et al. [33] analyzed the same SNP in 104 patients and 114 healthy controls from the state of Nuevo León, also finding no association. Apart from the fact that our study included a significantly higher number of participants (279 cases and 569 controls), and thus have more statistical power, the disparity of our results with these studies might reside in the populations analyzed. Although the subjects in the three studies were from México, the populations living in the three areas from which the samples were obtained could have differences in their ethnic background. In the study of Torres-Garcia et al., all participants (TB patients and controls) were from the Mazatecan ethnic group and were all living in the town called Temascal, a rural area in Oaxaca, and all were descendants of parents and grandparents born in the Mazatecan area in the state of Oaxaca, Mexico. Subjects of the Rosas-Taraco study have been residents of the state of Nuevo León for 2–3 generations and were from white and mestizo ethnic groups. In the northern part of Mexico, including the Nuevo León state, the population has overall less indigenous genes than in the central and southern parts of the country. Our population came from three different geographic areas of the state of Veracruz. Subjects from the northern part (Poza Rica) could have Totonacan ancestry (25% of our subjects from this area considered themselves as Totonacans), while in the central (Veracruz) and southern parts (Coatzacoalcos), less than 3% of the subjects declared themselves as belonging to an ethnic group, although most probably have some indigenous ancestry. It is known that there is great genetic diversity among indigenous ethnic groups in México [34], and although the study participants can be characterized as mestizos, genetic differences could exist among mestizos from different parts of the country. The low frequency of the minor alleles of these two polymorphisms in *TLR4* is also a factor that complicates a definitive conclusion as to their association with TB, since the number of homozygous individuals for the minor alleles is practically null in populations from Mexico and Hispanics living in the USA. Overall, only one homozygous individual for the minor allele in *TLR4* (D299G) was detected out of more than 1700 participants ([22, 27, 33] and this study).

In *in vitro* experiments, it has been demonstrated that heat shock protein Hsp 65 from Mtb activates TLR4-mediated proinflammatory pathways [35] and that the (D299G) polymorphism in TLR4 functionally affects the receptor, resulting in a blunted response to inhaled LPS in humans [14]. However, the influence of *TLR4* polymorphisms in susceptibility of humans to different infectious diseases has not been definitively established, suggesting that other environmental or genetic factors can potentiate or counteract the effects of these variations in *TLR4* for susceptibility to different conditions or diseases. In this regard, Ziakas et al. published a review of studies of the association of the two *TLR4* SNPs (D299G and T399I) and infections by numerous agents [36]. They concluded that the polymorphisms had been reported to be associated with increased, decreased, or no difference in susceptibility to infectious disease depending both in the population and the type of infection. Also, it should be kept in mind that the expression level, more than genetic variations in *TLR4*, has been proposed to be more critical for responsiveness to LPS [37]. Regarding the type of infection, it is interesting to note that the two SNPs in *TLR4* which we found associated with resistance to TB were reported to be associated with resistance to Legionnaires' disease [38] (caused by infection with *Legionella pneumophila*, an intracellular Gram-negative bacterium) and to be associated with a protective effect against leprosy caused by *Mycobacterium leprae* [39].

The SNP rs5743810 in *TLR6* is a nonsynonymous polymorphism that results in a serine to proline change of residue 249 in the extracellular domain of the receptor (S249P). This change has functional consequences, as it was shown that the variant allele (249P) was associated with lower NF-*κ*B signaling in response to diacylated lipopeptide (*p* = 0.019) or Mtb lysate (*p* = 0.026) in a HEK293 cell line reconstitution assay, compared with the ancestral allele (S249) [40]. This polymorphism has been studied for its association with TB, and results from these studies have been included in meta-analyses. Zhang et al. [41] included 4 case-control studies from two papers (1093 cases vs. 620 controls) and found that the minor allele (A) is associated with protection from TB (OR = 0.66, *p* = 0.04). Similarly, Schurz et al. [21] included four articles (7 studies) in their meta-analysis and also found a protective effect of the minor allele in the allelic model (A vs. G, OR = 0.66, *p* = 0.0001) and of the AA and AG genotypes in the heterozygote, homozygote, recessive, and dominant models. No studies have been reported of association of this SNP with TB in Mexican populations. Our results show no differences in allelic or genotypic frequency of this SNP between cases and controls (Table 2).

Human TLR10 is a receptor for which no ligands have been identified. Nevertheless, it has been shown that TLR10 is a modulatory receptor that can inhibit the TLR2-mediated release of proinflammatory cytokines [17]. An SNP in *TLR10* (different from the ones studied here) was found to be associated with TB in a Croatian population [42]. More recently, the influence of distinct polymorphisms in*TLR10* on susceptibility for TB has been reported [43, 44]. Although these studies did not analyze the SNPs included in our study, they nevertheless provide support for the possible participation of *TLR10* in TB. We studied two SNPs in this receptor for their possible association with susceptibility to TB: rs11466649 (A163S) and rs11466651 (V298I). These nonsynonymous SNPs, which are in strong linkage disequilibrium, have been studied for its association with TB only by Ma et al. [22] in three populations: African American, Caucasians, and Hispanics living in the state of Texas, USA. They found that the minor alleles in each of the SNPs are associated with increased susceptibility for TB (OR = 2.3, *p* = 0.003 for rs11466649 and OR = 2.07, *p* = 0.009 for rs11466651) in the Hispanic population. In our study population, however, none of the studied SNPs in *TLR10* were associated with the risk of developing active TB.

In summary, our results in a large cohort of 279 TB patients and 569 controls from the state of Veracruz, Mexico, has revealed that two cosegregating SNPs in *TLR4* are associated with a decreased susceptibility for active TB in this population. Our results suggest that the variants (299G) and (399I) in *TLR4* confer a protective effect against active TB disease in this population. Previously reported studies in different populations have found these polymorphisms were either not associated or associated with increased susceptibility for active TB. The reasons for these apparently contradictory results are not known but are probably related to the overall genetic background of the populations and/or to possible differences in pathogenicity of the circulating Mtb strains. In any event, and considering that despite efforts in several laboratories, it has not yet been possible to predict the phenotypic effect of *TLR4* variants in Mtb infections *in vivo* (reviewed in [45]), and the effect of polymorphisms in any given gene might be confounded by the overall genetic background of the studied population; our data contributes a piece of original information that should be taken into account in future meta-analyses and reviews.

## Figures and Tables

**Table 1:** Demographic characteristics of cases and controls.

	TB cases *n* = 279	Controls *n* = 659	OR (95% CI)	*p* value
Median age (years) (range)∗	37 (18-97)	38 (18-97)	—	0.13∗
Gender				
Female (%)	107 (38.4)	410 (72.1)	1	Reference
Male (%)	172 (61.6)	159 (27.9)	4.15 (3.03-5.68)	<0.001
Smoking (%)				
No	254 (91.1)	505 (88.8)	1	Reference
Yes	25 (8.9)	64 (11.2)	0.75 (0.46-1.29)	0.24
Alcohol use (%)				
No	232 (83.3)	455 (80.0)	1	Reference
Yes	47 (16.7)	114 (20.0)	0.80 (0.54-1.19)	0.20
Direct contact with patients				
No	21 (7.5)	46 (8.1)		Reference
Yes	257 (92.1)	516 (90.7)	1.09 (0.62 - 1.97)	0.89
Unknown	1 (0.4)	7 (1.2)		
Living in a rural area (%)				
No	205 (74.2)	447 (78.6)	1	Reference
Yes	74 (26.3)	122 (21.4)	1.32 (0.93-1.86)	0.10
Speak an indigenous language (%)				
No	229 (82.1)	514 (90.3)	1	Reference
Yes	50 (17.9)	55 (9.7)	2.04 (1.33-3.15)	<0.001
Educational level (%)				
Higher	15 (5.4)	133 (23.4)		
Complete high school	41 (14.7)	109 (19.2)		
Complete secondary school	71 (25.4)	127 (22.3)	1	Reference
Complete elementary school	100 (35.8)	145 (25.5)		
None	52 (18.6)	55 (9.7)	2.22 (1.66-2.97)	<0.0001

Abbreviations: TB: tuberculosis; OR: odds ratio; CI: confidence interval. ∗*p* value for age was calculated by Wilcoxon 2-sample rank-sum test. OR and 95% CI and *p* values for all other characteristics were assessed by using univariate logistic regression.

**Table 2:** Distribution of *TLR1*, *TLR2*, *TLR4*, *TLR6*, and *TLR10* allele and genotype polymorphisms in TB patients and controls of the state of Veracruz.

Gene SNP		TB patients *n* = 279		Controls *n* = 569		OR (95% CI)	*p* value^∗^
		*n*	Frequency	*n*	Frequency		
*TLR1*	CC	84	30.1	176	30.9	1	
N248S (rs4833095)	CT	135	48.4	277	48.7	1.02 (0.72- 1.48)	0.88
	TT	60	21.5	116	20.4	1.06 (0.68-1.64)	0.80
*Dominant*	TT+TC	195		393		1.03 (0.74-1.45)	0.84
	C	303	54.3	629	55.3	1	
	T	255	45.7	509	44.7	1.04 (0.84- 1.28)	0.82
*TLR2*	GG	279	100	568	99.8	1	
R753Q (rs5743708)	GA	0	0	1	0.2	—	ND
	G	558	100	1137	99.9	1	
	A	0	0.00	1	0.1	—	ND
*TLR4*	AA	275	98.6	537	94.4	1	
D299G (rs4986790)	AG	3	1.1	32	5.6	0.20 (0.06-0.69)	**0.01**
	GG	1	0.4	0	0	—	ND
*Dominant*	GG+AG	4		32		0.26 (0.09-0.77)	**0.02**
	A	553	99.1	1106	97.2	1	
	G	5	0.9	32	2.8	0.31 (0.09-0.81)	**0.01**
*TLR4*	CC	275	98.6	543	95.4	1	
T399I (rs4986791)	CT	4	1.4	26	4.6	0.35 (0.13-0.90)	**0.03**
	C	554	99.3	1112	97.7	1	
	T	4	0.7	26	2.3	0.29 (0.10-0.90)	**0.03**
*TLR6*	GG	236	84.6	488	85.8	1	
S249P (rs5743810)	GA	42	15.1	78	13.7	1.15 (0.76-1.74)	0.30
	AA	1	0.4	3	0.5	0.40 (0.04-4.29)	0.45
*Dominant*	AA+GA	43		81		1.22 (0.79-1.88)	0.38
	G	514	92.1	1054	92.6	1	
	A	44	7.9	84	7.4	1.08 (0.72-1.60)	0.70
*TLR10*	CC	192	68.8	374	65.7	1	
A163S (rs11466649)	CA	77	27.6	178	31.3	0.84 (0.60-1.18)	0.32
	AA	10	3.6	17	3.0	1.09 (0.45-2.57)	0.86
*Dominant*	AA+CA	87		195		0.86 (0.62-1.20)	0.38
	C	461	82.6	926	81.4	1	
	A	97	17.4	212	18.6	0.92 (0.70-1.20)	0.53
*TLR10*	CC	192	68.8	374	65.7	1	
V298I (rs11466651)	CT	77	27.6	178	31.3	0.83 (0.60-1.18)	0.33
	TT	10	3.6	17	3.0	1.08 (0.45-2.57)	0.86
*Dominant*	TT+CT	87		195		0.86 (0.62-1.20)	0.38
	C	461	82.6	926	81.4	1	
	T	97	17.4	212	18.6	0.92 (0.70-1.20)	0.53

Abbreviations: TB: tuberculosis; OR: odds ratio; CI: confidence interval; ND: not determined. ∗*p* values adjusted for gender, speaking an indigenous language, and educational level were determined by multivariate logistic regression.

**Table 3:** Analysis of haplotypes association with active TB.

Haplotype	Frequency	Case frequency	Control frequency	OR (95% CI)	*p* value
Chromosome 4					
CCCG	0.53	0.53	0.53	1.02 (0.83-1.26)	0.85
CCTG	0.21	0.22	0.21	1.02 (0.79-1.31)	0.88
TATG	0.17	0.16	0.17	0.98 (0.74-1.30)	0.91
CCTA	0.06	0.07	0.06	1.27 (0.83-1.93)	0.25
TACG	0.01	0.01	0.01	0.73 (0.20-2.15)	0.54
Chromosome 9					
AC	0.98	0.99	0.97	3.51 (1.36-11.53)	0.006
GT	0.02	0.01	0.02	0.35 (0.09-1.03)	0.043

Chromosome 4*: TLR10* A163S, *TLR10* V298I, *TLR1* N248S, *TLR6* S249P; chromosome 9*: TLR4* D299G, *TLR4* T399I.

## Data Availability

The data used to support the findings of this study are available from the corresponding author upon request.
